# Review of Xylanases: Sources, Engineering and Biotechnological Use

**DOI:** 10.3390/microorganisms14010127

**Published:** 2026-01-07

**Authors:** Elena Y. Pavlova, Danil O. Chesnokov, Nikolai M. Slynko, Andrey V. Zadorozhny, Yulia. E. Uvarova, Tamara M. Khlebodarova, Asya R. Vasilieva, Aleksandra A. Shipova, Natalia V. Bogacheva, Valeria N. Shlyakhtun, Anton V. Korzhuk, Ekaterina Y. Bukatich, Sergey E. Peltek

**Affiliations:** 1 Laboratory of Molecular Biotechnologies of the Federal Research, Center Institute of Cytology and Genetics, Siberian Branch of Russian Academy of Sciences, 630090 Novosibirsk, Russia; pavlovaey@bionet.nsc.ru (E.Y.P.); chesnokovdo@bionet.nsc.ru (D.O.C.); nslynko@bionet.nsc.ru (N.M.S.); evergreen@bionet.nsc.ru (A.V.Z.); uvarovaye@bionet.nsc.ru (Y.E.U.); vasilieva@bionet.nsc.ru (A.R.V.); a_ship@bionet.nsc.ru (A.A.S.); bogatcheva@bionet.nsc.ru (N.V.B.); shlyakhtun@bionet.nsc.ru (V.N.S.); korshuk_anton@bionet.nsc.ru (A.V.K.); bukatich@bionet.nsc.ru (E.Y.B.); 2 Kurchatov Genomics Center, Institute of Cytology and Genetics SB RAS, Akademika Lavrentyev Ave., 10, 630090 Novosibirsk, Russia; tamara@bionet.nsc.ru

**Keywords:** xylanase, xylan types, catalytic mechanisms, enzyme purification, recombinant expression, protein engineering, industrial biotechnology, biomass deconstruction

## Abstract

Xylanases (EC 3.2.1.8) are value-added enzymes essential for biomass deconstruction and are widely used in the pulp and paper, food, feed, and biofuel sectors. This review provides a comprehensive analysis of the current state and future prospects of xylanase research and application. It begins by examining the structural diversity of xylan substrates and the corresponding classification of xylanase enzymes, their catalytic mechanisms, and methods for their functional study, such as inhibitor analysis. The discussion then covers the challenges and methods involved in the purification of xylanases from complex biological mixtures. While natural microbial sources (fungi and bacteria) remain important, the limitations of wild-type (WT) strains for industrial production are highlighted. The review assesses the most common recombinant production systems, including *Escherichia coli*, *Bacillus subtilis*, and *Komagataella phaffii*, comparing their advantages for high-yield enzyme production. Finally, the paper focuses on protein engineering strategies as powerful tools for enhancing key enzyme properties (thermostability, specific activity, and pH tolerance). By integrating fundamental knowledge with applied technological approaches, this review underscores the critical role of xylanases in industrial biotechnology and identifies future research directions for their optimization.

## 1. Introduction

Xylan, the principal hemicellulosic polymer of plant cell walls, is among the most abundant renewable polysaccharides on Earth [1]. Its efficient conversion into value-added products is a key objective in biotechnology development. The main catalysts of this process are xylanases (EC 3.2.1.8), which hydrolyze β-1,4-glycosidic linkages in the xylan backbone. A broad spectrum of industrial applications—from juice clarification and enhancement of baked goods to pulp production and biofuel manufacture—drives a steadily increasing demand for these highly efficient and environmentally friendly biocatalysts.

Xylanases obtained from native producers (fungi and bacteria) often fail to meet industrial production requirements; they are frequently characterized by low enzyme yields and complex purification procedures [2,3]. A solution to these issues has been the heterologous expression of xylanase genes in recombinant microbial hosts. This strategy enables not only high-level production of a single, defined enzyme but also targeted modification of its properties [3].

A diverse suite of expression platforms is available today for recombinant xylanase production, each with its own benefits and limitations. These encompass bacterial and yeast systems in addition to filamentous fungi [2]. The optimal host selection is dictated by factors including the degree of post-translational processing required, the target mode of protein localization (intracellular accumulation versus high-efficiency secretion), and the scalability of the production workflow.

Further optimization of xylanase functional properties is achieved through protein engineering to endow the enzymes with desired attributes, such as increased thermostability, robustness at alkaline pH, and enhanced catalytic activity

The aim of this review is to provide a systematic analysis of recent advances and emerging directions in the production of recombinant xylanases. The work covers, in detail, the structural diversity of xylans and xylanase classification, the main enzyme sources and heterologous expression systems, protein engineering methods aimed at improving xylanase properties and its industrial applications.

The conducted analysis will elucidate key trends and identify the most promising avenues for future research aimed at developing high-performance biotechnological solutions based on xylanases.

## 2. Industrial Applications of Xylanase

Enzymatic hydrolysis of xylan is an important area in biotechnology, with applications in various industries such as food production, pulp and paper, textiles, animal feed, waste treatment, and biofuel production. Additionally, xylanases are significant for the production of rayon, cellophane, and some chemicals such as cellulose esters, which are derived from dissolved pulp, and purified fibers of other carbohydrates [2,4,5].

Enzymes possessing unique properties, such as thermostability and pH stability, are particularly valuable. For example, thermo-alkali stable xylanases are in demand for the pulp and paper industry for bio-bleaching [6], and in the textile industry for desizing and scouring processes [5,7]. Thermostable xylanases also find application in bioethanol production, where the process occurs at high temperatures and pressures [8].

**Figure 1:**
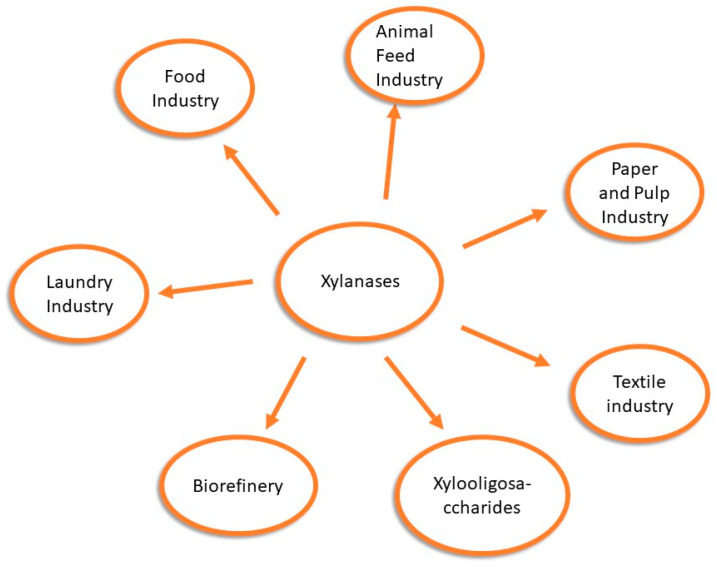
Examples of xylanases applications in industry.

Examples of xylanases applications in industry are shown in Figure 1.

### 2.1. Food Industry

Xylanases are widely utilized in the food industry. In bakery applications, xylanases improve bread quality by modifying the gluten network structure, reducing hardness, increasing specific volume, and delaying staling [9,10]. These effects are primarily due to the enzyme’s ability to enhance the solubility of wheat arabinoxylans [5,11]. Moreover, the products of arabinoxylan degradation, such as arabino-xylooligosaccharides, are presumed to be health-beneficial, thus increasing bread’s nutritional value [6,7].

Another significant application of xylanases is in fruit juice clarification. Raw fruit juices contain polysaccharides—including cellulose, hemicellulose, starch, pectin, and surface-bound lignin—that can impair juice quality by causing haze and high viscosity [5]. Enzymatic clarification improves the clarity, sensory attributes, and storage stability of fruit juices. Currently, physical, chemical, and enzymatic methods are employed worldwide for juice clarification [12,13].

Additionally, xylanases play a vital role in the bioconversion of xylan into value-added products such as xylitol, which is widely used as a sweetener in soft drinks, candies, ice cream, chewing gum, and pharmaceutical formulations. Xylitol also serves as a natural sweetener in dental care products like toothpaste [3].

### 2.2. Animal Feed Industry

The application of xylanases in animal feed manufacturing offers significant benefits by the degradation of hemicelluloses, which results in decreased feed viscosity and enhanced nutrient bioavailability. Specifically, xylanases aid in hydrolyzing starch polysaccharides within fiber-rich and barley-based feeds. Pre-treating agricultural silage and grains with xylanases has been demonstrated to elevate nutritional value and facilitate improved digestive efficiency in ruminants [3]. Furthermore, xylanase supplementation has been shown to promote weight gain and improve feed conversion ratios in monogastric animals, attributable to the increased digestibility of arabinoxylans [14].

### 2.3. Paper and Pulp Industry

#### 2.3.1. Bio-Bleaching

The bleaching process involves the removal of lignin from chemical pulps to produce products with increased brightness or whiteness [5,15,16].

This process is necessary due to the residual lignin and its derivatives present during pulping, which impart a characteristic brown coloration to the cellulose. The intensity of this coloration depends on both the quantity and the chemical state of the remaining lignin. The primary goal of pulp bleaching involves the degradation, modification, or solubilization of lignin, colored organic compounds, and other undesirable residues associated with the fibers. Bio-bleaching, which utilizes enzymes or ligninolytic microorganisms, represents a more environmentally sustainable alternative to conventional bleaching processes [2].

Xylanases can hydrolyze xylan linked to cellulose and lignin within pulp fibers. Disruption of xylan facilitates the separation of these components, enhances fiber wall swelling, and improves lignin removal [5,15]. The application of xylanases resulted in reduced chemical consumption, lower concentrations of chlorinated organics in waste effluents and pulp, and a decreased chlorine requirement [17]. Moreover, enzyme treatment can enhance fiber bonding forces and improve paper strength properties [18].

#### 2.3.2. Deinking of Waste Paper

The removal of ink from waste paper is a crucial step in the recycling and reuse process. Traditional chemical methods, employing chlorine or chlorine-based compounds, ClO^−^, NaOH, Na_2_CO_3_, H_2_O_2_, and Na_2_SiO_3_, have been extensively used. However, these methods produce toxic effluents that require elaborate treatment prior to disposal, posing environmental concerns [5].

Bio-bleaching with xylanases offers a promising alternative, significantly reducing the reliance on chemical bleaching agents while maintaining paper quality. The enzymatic deinking process often involves a mixture of enzymes, including xylanases, cellulases, pectinases, and lipases, which facilitate ink removal by cleaving the chemical bonds between ink particles and paper fibers or by altering the surface characteristics of the paper [19].

### 2.4. Textile Industry

Microbial enzymes have revolutionized the textile industry by replacing harmful chemicals with environmentally friendly alternatives, improving such processes as desizing, scouring, dyeing, and finishing, and contributing to water conservation and reduced environmental pollution. Enzymes such as amylases, pectinases, cellulases, and oxidoreductases have demonstrated their ability to substitute hazardous chemicals at key stages of textile processing, including sizing, cleaning, bleaching, and finishing. These biocatalysts not only enhance fabric quality by increasing softness, improving appearance, and prolonging durability, but also substantially reduce environmental impact. Moreover, enzymes catalyze specific reactions with high selectivity, ensuring precise textile processing while preserving fabric integrity [20,21,22].

Despite their potential, enzymatic processing methods face several challenges, including substrate specificity and sensitivity to environmental factors such as pH, temperature, and the presence of inhibitors, as well as limitations in compatibility with certain surfactants and other additives. In addition, the high costs associated with enzyme production and purification remain a limiting factor, although recent advances in recombinant DNA technology and protein engineering are progressively lowering these barriers [20].

The enzymatic activity of xylanase in degrading xylan within hemicellulose has found significant application in the textile industry, notably in the degumming of ramie fiber textiles [23] and in desizing processes [24]. Xylanase selectively hydrolyzes xylan within gummy substances, enabling effective degumming with minimal damage to the fibers. When combined with a suitable amount of dilute alkali, xylanase facilitates efficient degumming under reduced temperature and pressure conditions, thereby preserving fiber integrity and quality. However, the application of xylanase in degumming, particularly for plant fibers such as ramie, is constrained by the limited alkaline stability of available enzymes [23].

### 2.5. Xylooligosaccharides

Xylooligosaccharides (XOS), oligomers formed by non-digestible xylose monomers, are increasingly utilized in various areas including biotechnology, pharmaceuticals, food, and animal feed [5]. Their principal feature is a prebiotic effect, wherein it promotes the growth of beneficial gut bacteria even at low daily dosages (1.4–2.8 g/day) [25]. Additionally, XOS have been shown to enhance lipid metabolism, reduce the expression of proinflammatory mediators, and exhibit antioxidant and antimicrobial activities [2,26]. They contribute to cholesterol reduction, inhibit starch retrogradation, and improve calcium bioavailability, thereby enhancing the nutritional qualities of food. Moreover, XOS are non-toxic and are certified as Generally Recognized as Safe (GRAS) [25]. Consequently, they are used in beverage products such as tea, coffee, soy milk, and dairy items [27]. In pharmaceuticals, XOS have shown potential as immunomodulators, anti-cancer agents, antimicrobials, antioxidants, anti-allergy substances, anti-inflammatories, and lipid-lowering agents. They are also employed in phyto-pharmaceuticals and animal feed applications, exhibiting growth-regulatory effects in aquaculture and poultry [5].

XOS have been extensively utilized in industrial processes, particularly in chemical manufacturing and biofuel production [28]. For example, in study [29], xylose monomers obtained through xylan degradation in sunflower crops using xylanase served as substrates for fermentation with *Trichoderma atroviride* strains to produce biobutanol.

### 2.6. Biorefinery

The depletion of non-renewable energy resources is progressing at alarming rates, posing a significant risk of an impending energy crisis [8]. Consequently, there is an increasing demand for renewable energy sources, including biofuel production. Agricultural waste biomass emerges as an ideal feedstock due to its low cost and high availability [30]. Efficient conversion of lignocellulosic biomass (LCB) into fuel-grade ethanol has become a global priority aiming to provide an environmentally sustainable and cost-effective fuel for the transportation sector [5].

Recently, the biological conversion of lignocellulose into ethanol has attracted considerable research interest due to its environmental benefits and efficiency. Lignocellulose comprises lignin, cellulose, proteins, and pectins in varying proportions [31]. Enzymatic hydrolysis of LCB, which is environmentally friendly, is considered a preferred method for biomass deconstruction. Complete hydrolysis of lignocellulose requires a suite of enzymes, including endoglucanases (EC 3.2.1.4), β-glucosidases (EC 3.2.1.21), endo-1,4-β-xylanases (EC 3.2.1.8), and β-xylosidases (EC 3.2.1.37) [8].

The delignification process releases hemicellulose and cellulose from their complex with lignin, enhancing enzyme accessibility [8]. Xylanases catalyze the breakdown of β-1,4-xylan into xylooligosaccharides, xylobiose, and xylose units [27]. These carbohydrates are subsequently hydrolyzed into simple monosaccharides, which can be fermented into ethanol, representing a sustainable energy pathway [8].

### 2.7. Laundry Industry

At present, various enzymes (e.g., amylases, cellulases, proteases, and lipases) are widely used in household detergents. However, these enzymes do not remove all types of soil commonly found on textile materials. Different stains originating from plants (grass, spinach, carrot, cherry, grape, etc.) and stains from beverages derived from plant raw materials (wine, beer, fruit juices) cannot be completely eliminated by detergents alone. The pigments contained in such plant materials are associated with fibrous components that constitute the major part of the plant cell wall. These pigments are often difficult to remove, as they are predominantly represented by non-starch polysaccharides (pectin, cellulose, hemicellulose). Therefore, efficient removal of stains from textile materials requires the use of appropriate enzymes. Typically, detergents contain a bleaching agent that is capable of decolorizing stains but not removing them. It should also be noted that such agents may cause damage to the fabric [32].

Despite the fact that research on the use of xylanases in the textile industry for fabric cleaning is currently very limited, there are already promising examples of xylanases being applied as biological bleaching agents. For instance, in [32], a xylanase from *Aspergillus niger* was successfully developed as a detergent additive to improve washing performance.

## 3. Xylan Substrates and Xylanase Enzymes: Families, Catalytic Mechanisms, and Inhibitor Analysis

Lignocellulose is a complex natural composite composed of three main components: cellulose (40–60%), hemicellulose (20–30%), and lignin (15–25%). Cellulose and hemicellulose are polysaccharides, while lignin is a three-dimensional aromatic polymer. Together, these components constitute the dry matter of plant biomass, providing strength and integrity to cell walls [5]. The term “hemicellulose” was first introduced by Schulze [11] to describe the fractions extracted from plant material with dilute alkalis.

Xylan is the main component of hemicellulose and the second most abundant polysaccharide in nature, accounting for approximately one-third of the Earth’s renewable organic carbon [7]. In a broader sense, hemicelluloses also include xyloglucan (a heteropolymer of D-xylose and D-glucose), glucomannan (a heteropolymer of D-glucose and D-mannose), galactoglucomannan (a heteropolymer of D-galactose, D-glucose, and D-mannose), and arabinogalactan (a heteropolymer of D-galactose and arabinose). Xylan is primarily localized in the secondary cell walls, where it forms a structural framework together with cellulose and lignin [6]. These three components interact via covalent and non-covalent bonds, with xylan situated at the interface between cellulose and lignin, providing fiber cohesion and structural integrity to the cell wall [9].

### 3.1. Structural Diversity and Distribution of Xylans

Xylan is a complex, typically highly branched polysaccharide whose structure varies depending on the plant type. Its backbone consists of 1,4-linked β-D-xylopyranosyl residues, which are partially substituted with various side groups, including glucuronopyranosyl, 4-O-methyl-D-glucuronopyranosyl, α-L-arabinofuranosyl, acetyl, feruloyl, or p-coumaroyl residues [6,12].

Linear, nearly unsubstituted xylans have been identified in esparto grass (*Stipa tenacissima*) [14], tobacco (*Nicotiana tabacum*) [15], and some seaweeds [18]. In hardwood species, xylan is primarily present as O-acetyl-4-O-methylglucuronoxylan, whereas in softwoods, it exists as arabino-4-O-methylglucuronoxylan. Arabinoxylans are the predominant form in grasses and annual plants. The degree of polymerization of xylans also varies: in hardwoods, it is approximately 150–200 β-xylopyranose units, compared to 70–130 in softwoods.

### 3.2. Principal Xylan Families

Xylans are classified into five major families [11]:

Arabinoxylans are characterized by side chains of single α-L-arabinofuranosyl residues. Cereal arabinoxylans show 2-O- and 3-O-monosubstitution patterns, along with disubstituted (2-O-, 3-O-) xylosyl residues.

Glucuronoxylans are characterized by having α-D-glucuronic acid or its 4-O-methyl derivative as their only side chain.

Glucuronoarabinoxylans are characterized by the presence of both α-D-glucuronic acid (or 4-O-methyl-α-D-glucuronic acid) and α-L-arabinose as side-chain substituents.

Galactoglucuronoarabinoxylans are characterized by the presence of terminal β-D-galactopyranosyl residues on oligosaccharide side chains; they are typically found in perennial plants.

Homoxylans represent a rare class of natural polysaccharides consisting entirely of xylosyl residues.

Microheterogeneity in branching patterns exists within each xylan family. The side chains determine the solubility, spatial configuration, and reactivity of xylan, thereby influencing the efficiency of its enzymatic hydrolysis [6]. The endospermic arabinoxylans of annual plants, also known as pentosans, exhibit greater solubility in water and alkalis compared to the xylans of perennial plants, which is attributed to their more highly branched structure [26].

Historically, the structural analysis of lignocellulosic polysaccharides relied on the chemical hydrolysis of xylans. This process serves as a model system for illustrating the mode of action of xylanases. Under mild acidic or alkaline conditions, the β-1,4-glycosidic bonds of the main xylose backbone are cleaved, and side substituents are partially removed. This results in the formation of water-soluble xylan derivatives and xylooligosaccharides. These products are characterized by high viscosity and an ability to form stable colloidal systems, which has led to their use as thickeners, stabilizers, and film-forming agents [3,33].

Chemical hydrolysis is characterized by low selectivity, frequently resulting in the destruction of functional groups and the degradation of sugars into furan derivatives. In contrast, the enzymatic hydrolysis of xylans, catalyzed by xylanases, proceeds under mild and specific conditions, yielding well-defined oligomers without by-products. The catalytic efficiency of these enzymes can exceed the rate of non-enzymatic hydrolysis by many orders of magnitude, up to 10^17^-fold [27]. Thus, xylanases represent a biological analogue of chemical hydrolysis while acting with greater precision and preserving polymer structural integrity, making them a key tool in the biotechnological processing of plant biomass.

### 3.3. Types and Families of Xylanases

Xylanases belong to the class of hydrolases that catalyze the hydrolysis of the β-1,4-glycosidic bond in xylans, resulting in a sugar hemiacetal and the corresponding aglycone. Based on their mode of action, they are classified into endoxylanases, which cleave internal bonds within the polysaccharide chain, and exoxylanases, which release xylose residues from the chain ends. This classification is based on substrate specificity and the type of catalyzed reaction but does not reflect the structural diversity of the enzymes. Consequently, functionally similar enzymes from different organisms, as well as distinct enzymes within the same organism, can share the same EC number (Enzyme Commission of IUBMB) [34].

According to the international enzyme classification system, hydrolases belong to class EC 3. Enzymes of this class are divided into 13 subclasses based on the type of bond they hydrolyze. O-Glycoside hydrolases fall into subclass EC 3.2, and xylanases are categorized under EC 3.2.1.x. The numerical identifier “x” (from 1 to 207) is assigned based on the carbohydrate moiety of a glycoside structure and substrate specificity.

The original classification of xylanases, which segregated them into two groups based on molecular mass (below or above 30 kDa) and isoelectric point (pI) (basic or acidic), is now largely obsolete for modern enzymes [5]. This limitation prompted the development of a classification system founded on the primary structure of their catalytic domains. Enzymes are now grouped into families based on sequence homology, a method that serves as the current standard for all glycosyl hydrolases (GHs). GHs are the most extensive group of enzymes that catalyze the hydrolysis of glycosidic bonds between carbohydrate moieties, as well as between a carbohydrate and a non-carbohydrate moiety. This sequence-based classification reveals evolutionarily conserved protein structures, allowing for the grouping of families into clans. To date, there are 14 different clans (from GH-A to GH-N), with most clans comprising two or more families [3,35].

Information on xylanases and their classification into families is available in the CAZy (Carbohydrate-Active Enzymes) database, a resource dedicated to enzymes involved in the synthesis and metabolism of diverse carbohydrates [36]. Xylanases are subdivided into 13 families; however, only families GH10 and GH11 exhibit exclusive endo-β-xylanase activity. These two families are distinguished by their amino acid sequences, catalytic properties, substrate specificity, three-dimensional structures, and mechanisms of action [2,3,35]. It is noteworthy that families harboring xylanase activity often include bifunctional enzymes, such as those with endoglucanase, licheninase, or arabinofuranosidase activities. Therefore, xylanase activity is not confined to GH10 and GH11 but is also present in other families, including GH5, GH7, GH8, GH16, GH43, GH52, and GH62 [3,35,37]. Xylanases from families GH10 and GH11 hydrolyze glycosidic bonds via acid-base catalysis through a double displacement mechanism, which preserves the anomeric configuration at the cleavage site [3].

Among the well-characterized xylanase families, GH10 xylanases (clan GH-A) feature a conserved (α/β)8-barrel structure with a catalytic cleft encompassing 4–7 xylose-binding subsites [3,38]. The catalytic center comprises two glutamate residues, one acting as a nucleophile and the other as an acid-base catalyst. The enzymatic mechanism and the amino acids involved in catalysis are conserved, forming a catalytic domain of 250–400 amino acids. The pI values of xylanases from this family are generally alkaline (8.0–9.5), although variants with acidic pI are also found. Most substrate-binding subsites in these xylanases are highly conserved; however, differences in affinity between subsites significantly influence the mode of action and substrate preferences [3,39,40]. It has been proposed that intra- and intermolecular interactions, such as hydrogen and disulfide bonds, overall folding compactness, stabilization of N- and C-terminal regions, and a low B-factor, contribute to the enhanced thermostability observed in this family [3,41]. This family includes endo-1,4-β-xylanases and endo-1,3-β-xylanases (EC 3.2.1.32). GH10 members can hydrolyze the aglycon bond of aryl-β-glycosides of xylobiose and xylotriose and exhibit high activity towards short xylooligosaccharides, indicating the presence of small substrate-binding sites [2].

Additionally, more detailed consideration should be given to the molecular structures underlying the interactions between GH10 xylanases and various substrates. In this study [42], theoretical complexes were constructed by modeling the incorporation of xylooligosaccharides (XOS) into the active site cleft of TmxB, a highly thermostable xylanase derived from *Thermotoga maritima*. The results indicated that subsites ranging from −2 to +2 are essential for substrate recognition. The +2 subsite maintained the xylosyl residue via hydrogen bonding networks rather than through conventional hydrophobic stacking interactions at the aglycone subsite in GH10 xylanases. Conserved amino acids were distributed across the subsites as follows: −1 (His104, Trp108, Asn152, Glu153, Gln228, His230, Glu259, and Trp308), −2 (Glu67, Asn68, Lys71, Gln111, and Trp300), as well as the subsites +1 (Tyr196) and +2 (Ser197 and Glu199).

**Figure 2:**
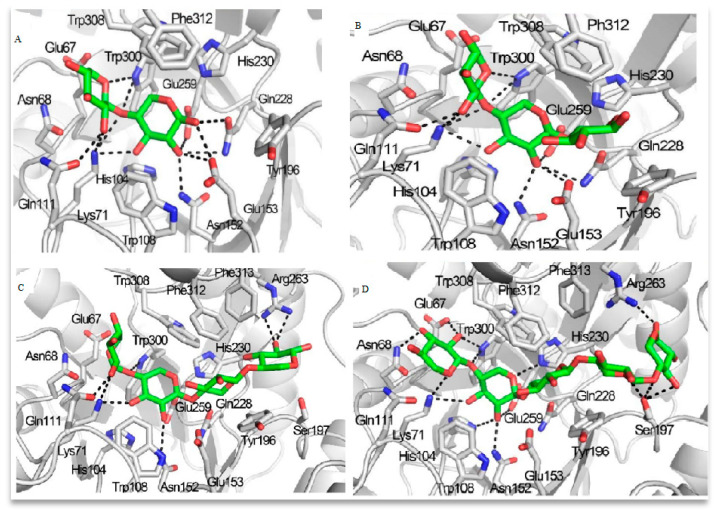
Docking structure of TmxB in complex with xylanes. (**A**) Interaction details between TmxB and xylobiose molecule in the docking structure; (**B**) interaction details between TmxB and xylotriose in the docking structure; (**C**) interaction details between TmxB and xylotetrose; (**D**) interaction details between TmxB and xylopentose. The putative hydrogen bonds are indicated by black dashes. The carbon atoms of amino acids and oligoxylanes are shown in gray and green, respectively. Adapted from [42].

**Figure 3:**
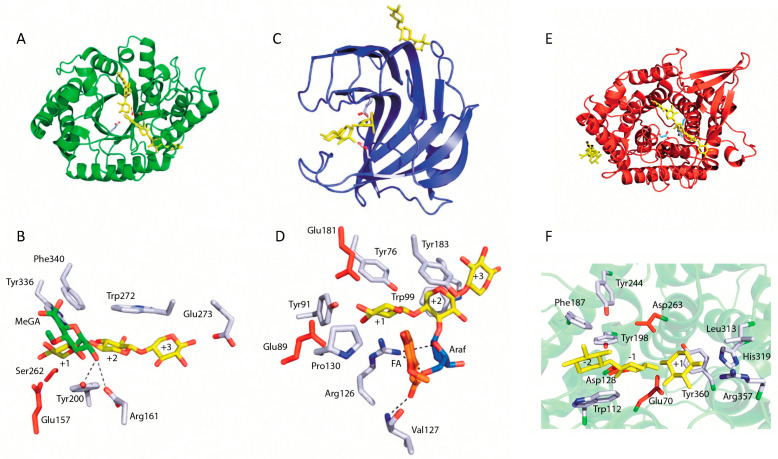
(**A**) Overall structure of GH10 *Cellvibrio mixtus* xylanase (PDB 1UQY). The catalytic domain has a (β/α)8-barrel fold and binds substrate (yellow) in the active site cleft. The catalytic residues in the active-site cleft are indicated. (**B**) Binding of 4-O-methylglucuronic acid (MeGA, green) in the active site of GH10 xylanases. Catalytic residues are indicated in red. Amino acids that are important for binding of xylose residues in the subsites are indicated. MeGA makes direct interactions with the protein by hydrogen bonds with Tyr200 and Asp161. (**C**) Overall structure of GH11 Bacillus subtilis xylanase A (PDB 2QZ3). The catalytic domain has a β-jelly roll fold and binds substrate (yellow) in the active site cleft and at the surface. (**D**) Binding of feruloyl-arabinose α-1,3 linked to the central xylose of xylotriose (XA5f3X) (yellow) in the +2 sub-site of a GH11 xylanase (PDB 2VGD). Catalytic residues are indicated in red. Arabinose (Araf) (blue) and ferulic acid (FA, orange) make hydrogen bonds with Arg126 and Val127. Amino acids that are important for binding of xylose residues in the +1, +2, and +3 subsites are indicated. (**E**) Overall structure of GH8 *Pseudoalteromonas haloplanktis* xylanase (PDB 2B4F). The catalytic domain has a (α/α)6-barrel fold and binds substrate (yellow) in the active site cleft and at the surface. (**F**) Detail of the active site of GH8 *Bacillus halodurans* Rex (PDB 1WU5 and 1WU6). Amino acids that are important for substrate binding and hydrolysis are indicated. The catalytic residues (Glu70, Asp263, and Asp128) are colored red. Leu318 and His319 block the aglycon side of the active-site cleft at subsite +2. Adapted from [38].

Figure 2 and Figure 3 illustrate the models of substrate interactions with the active sites of the respective enzymes.

Xylanases from family GH11 belong to clan GH-C [43]. They exhibit exceptional specificity for polymeric, insoluble xylose-containing substrates. The structure of GH11 xylanases is highly homologous and consists of a single major α-helix and two extensive β-sheets that form a jelly-roll fold. Key features include an 11-residue loop bridging two β-strands and a long substrate-binding cleft housing the active site. Catalysis is mediated by two conserved glutamate residues functioning as a nucleophile and an acid-base catalyst [44,45]. The catalytic amino acids and the mechanism of action of GH11 family enzymes are conserved and comprise a domain of 180–200 amino acids that folds into a β-sheet conformation [3].

This division reflects structural and functional differences: GH10 enzymes possess a broader active site and are capable of degrading heavily substituted xylans, whereas GH11 enzymes predominantly target linear regions of the polymer. Intrafamily homology is supported by crystallographic and kinetic data, establishing the CAZy classification as a universal basis for modern xylanase nomenclature [35].

**Figure 4:**
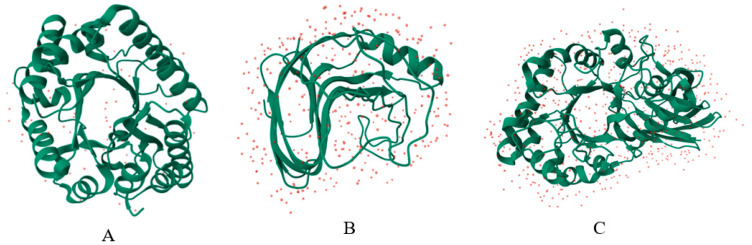
Structures of some types of xylanases from different families. (**A**)—Xylanase 2EXO exhibits the characteristic GH10 family structure, a (α/β)8-barrel, with alpha-helices on the exterior and beta-strands on the interior. (**B**)—Xylanase 1H1A from the GH11 family displays a typical architecture consisting of an alpha-helix and two extended beta- sheets. (**C**)—Xylanase 1NOF from the GH5 family [15]. Water molecules are represented by orange dots.

The GH5 family (clan GH-A) is the largest group of GH, encompassing enzymes with various activities, including endo-1,4-β-xylanase activity. Enzymes in this family hydrolyze the β-1,4-xylan chain at a specific site, which is determined by the position of the α-1,2-linked glucuronate fragment. Structural analysis of GH5 xylanases has revealed that the catalytic domain adopts a (β/α)8-barrel fold [3,46]. GH8 family xylanases, classified within clan GH-M, also exhibit β-1,4-xylanase activity and possess a (α/α)6-barrel structure, composed of six inner and six outer α-helices [3,47]. Figure 4 illustrates the structures of selected xylanases from families GH10, GH11, and GH5.

Enzymatic hydrolysis of xylans can proceed via retention (families GH5, GH7, GH10, GH11) or inversion (GH8, GH43) of the anomeric center of the restoring sugar [4]. In the first group of enzymes, the reaction follows a double-displacement mechanism involving two glutamic acid residues. In this process, one residue acts as a general acid, protonating the leaving group, while the other serves as a nucleophilic catalyst, forming a covalent α-glycosyl intermediate. In the second step, the first residue functions as a base, activating a water molecule, which results in a second displacement and the restoration of the original (β) configuration at the anomeric center [35,48].

Enzymes of the GH8 and GH43 families operate via an inverting mechanism, in which catalytic residues of glutamic and aspartic acids participate. One carboxyl group facilitates acid catalysis by protonating the leaving group, while the other activates a water molecule, inducing a nucleophilic attack on the anomeric carbon and resulting in inversion of configuration. The spatial arrangement of the catalytic residues in these enzymes positions the water molecule between the anomeric carbon and the general base [35].

The characteristics of enzyme adaptation to various types of plant substrates arise from evolutionary differences, notably between the GH10 and GH11 families. GH10 xylanases, predominantly found in bacteria, are specialized for degrading heterogenic, heavily substituted xylans that form during the partial decomposition of LCB. In contrast, GH11 xylanases, mainly present in fungi, are more effective in hydrolyzing linear arabinoxylans present in cereal cell walls [35,36]. These features determine their biotechnological applications: GH10 xylanases are widely used in LCB bioconversion processes, whereas GH11 enzymes are utilized in the food, feed, and paper industries, where controlled modification of polysaccharide structures without complete degradation is required.

Recent advances in structural biology have substantially expanded our understanding of xylanase architecture, substrate recognition mechanisms, and catalytic dynamics. State-of-the-art high-resolution techniques, including cryogenic X-ray crystallography, cryo-electron microscopy (cryo-EM), advanced nuclear magnetic resonance (NMR) spectroscopy, and (small-angle X-ray scattering) SAXS, in addition to classical protein crystallography, make it possible to study xylanases under conditions close to their native environment, including flexible domains, oligomeric states, and transient enzyme–substrate complexes [49,50].

Cryogenic X-ray crystallography (cryocrystallography) has recently emerged as a powerful tool for elucidating the structures of carbohydrate-active enzymes, including xylanases. Cooling protein crystals to 100 K reduces thermal motion and affords sub-ångström resolution, which cannot be achieved at room temperature. This approach provides access to the structural characterization of solvation shells, weak interactions, and catalytically relevant protonation states that are essential for understanding the catalytic mechanism characteristic of GH10 and GH11 xylanases.

The potential of this approach is well illustrated by the structure of a thermostable GH11 xylanase from *Thermoascus aurantiacus*, determined at 0.89 Å (100 K) and 1.11 Å (293 K) resolution [51].

### 3.4. Inhibition Analysis

Inhibition analysis represents a refined and highly informative tool for probing xylanase structure and function. It facilitates the investigation of the catalytic mechanism, the determination of substrate affinity via inhibition constants, and the identification of functional residue roles and substrate-binding types. The inhibitors employed in this analysis fall into three main categories:

#### 3.4.1. Active Site-Specific Inhibitors

1-(4-Azonia-4,4-dimethylpentyl)-3-ethylcarbodiimide iodide (EAC) is a water-soluble carbodiimide derivative similar in action to classical EDC (l-ethyl-3[3-(diethylamino)-propyl]-carbodiimid) but exhibits higher reactivity and stability in aqueous buffers. The use of EAC demonstrated the involvement of carboxyl groups (glutamic acid residues) in the catalysis of xylanase A (GH10) from Schizophyllum commune. Treatment with EAC led to rapid enzyme inactivation. This inactivation was prevented in the presence of xylan, indicating the location of the modified residue within the active site. The modification product was identified as an N-acylurea derivative of glutamic acid, confirming the crucial catalytic role of Glu127 [52].

Similarly, N-bromosuccinimide completely inactivated the active site of a xylanase from a thermophilic Bacillus strain. This observation led the authors [53] to conclude that tryptophan and cysteine residues play key roles in the enzyme’s active site.

#### 3.4.2. Substrate Analogs and Pseudosubstrates

Azosugars and their derivatives function as structural mimetics of the transition state [54]. And in this case, the most effective compounds are imidazole derivatives, exhibiting Ki values in the range of 10^−7^ to 10^−8^ M. This efficacy is attributed to imidazole’s ability to mimic the electron density of the oxocarbonium transition state and to participate in hydrogen bonding interactions with catalytic glutamates.

These inhibitors help to determine the inhibition mechanism (competitive, non-competitive, mixed) and quantify the kinetic parameters, the Michaelis constant and the maximum reaction velocity.

The activity of xylanases can be inhibited by natural oligosaccharides. The findings of the molecular modeling and docking experiments lend support to the hypothesis that three xylanase enzymes, which are taxonomically distinct and exhibit disparate sequences and three-dimensional structures, are similarly inhibited by natural oligosaccharides. Non-branched (linear) oligosaccharides exert a competitive inhibitory effect on the activity of xylanases. Even at low concentrations, branched oligosaccharides inhibit xylanase activity in a non-competitive manner. Oligosaccharides comprising a minimum number of subunits (triose, tetrose, and pentose) exert a particularly potent inhibitory effect on the activity of xylanases. The new results offer a molecular rationale for the findings reported in previously published scientific and industrial communications in peer-reviewed journals [55].

#### 3.4.3. Metal Cation Inhibitors

The inhibition of xylanases by metal cations is generally attributed to their ability to form complexes with functional groups in the active site, such as carboxyl and thiol groups. Ions like Hg^2+^, Cu^2+^, and Pb^2+^ cause irreversible loss of activity, whereas Zn^2+^ and Fe^3+^ exhibit partial inhibition. Conversely, Ca^2+^ and Mg^2+^ can stabilize the structure of GH10 xylanases at low concentrations but act as inhibitors at higher concentrations. Thus, the effect of a metal cation is determined by its position in the hardness series and its affinity for the donor atoms in the active site [35,56].

In most xylanases from the GH10 and GH11 families, catalytic activity can proceed independently of metal cofactors. Consequently, metal ion-induced inhibition underscores their structural rather than catalytic function within these enzymes.

#### 3.4.4. Proteinaceous Inhibitors

A distinct category is represented by proteinaceous xylanase inhibitors, known as Xylanase Inhibitor Proteins (XIPs). These compounds serve a protective function in plants, preventing the degradation of the cell wall by microbial enzymes. These low-molecular-weight proteins (20–45 kDa) bind to the active site of fungal and bacterial xylanases, forming reversible 1:1 complexes and blocking the enzyme’s access to its substrate [57,58]. The most extensively studied families of these inhibitors are TAXI (*Triticum aestivum* xylanase inhibitor) and XIP. TAXI-type proteins, found in wheat and rye, are highly specific for fungal GH11 xylanases, whereas XIP-type proteins inhibit both GH11 and GH10 enzymes of bacterial origin. TLXI proteins, which are related to PR-5 proteins like thaumatin, block the active site and alter the enzyme’s conformation, acting as non-competitive inhibitors [59,60].

Inhibition by proteinaceous factors is of significant biological importance as it reflects the co-adaptation between pathogenic microorganisms and host plants: plants produce XIPs in response to infection, while fungi evolve xylanase variants with reduced susceptibility. The inhibition constants for these complexes are in the nanomolar range (K_i_ ~10^−8^–10^−7^ M).

From a practical standpoint, the presence of natural proteinaceous inhibitors explains the reduced efficacy of supplemental xylanases in cereal processing and must be considered during the enzymatic treatment of plant biomass.

## 4. Sources of Natural Xylanase-Producing Microorganisms

Xylanases are produced by a wide range of organisms, including bacteria, archaea, fungi, actinomycetes, insects, algae, plants, protozoa, mollusks, and nematodes, encompassing extremophiles, mesophiles, thermophiles, as well as anaerobic and aerobic microorganisms [18,37]. Xylanase-producing microorganisms were isolated from diverse environments, such as compost, saline lakes, and the rumen of ruminants, among others. The ecological niches inhabited by xylanase producers are similarly diverse, including marine and terrestrial habitats where plant biomass decomposes, as well as mesophilic and thermophilic environments [37,61].

### 4.1. Fungal Sources of Xylanases

Fungi are the main producers of xylanases, largely due to their capacity to secrete significant quantities of the enzyme into the extracellular space. Xylanases from fungi display higher activity compared to those from yeasts and bacteria [17]. Genera typically associated with xylanase production include *Aspergillus*, *Coriolus*, *Fusarium*, *Phanerochaete*, *Trichoderma*, *Pichia*, and *Penicillium* [37,61].

Filamentous fungi are of particular interest in industrial applications because they produce extracellular xylanases along with other auxiliary enzymes that participate in the hydrolysis of xylan into its monomeric and oligomeric components [37,62].

### 4.2. Bacterial Sources of Xylanases

Xylanases obtained from bacterial and actinomycete sources demonstrate activity across a pH range of 5 to 9. It has also been demonstrated that their activity is optimal in a wide temperature spectrum, from 35 °C to 60 °C. In industrial contexts, bacterial xylanases are more desirable owing to their superior pH and thermal stability compared with fungal counterparts. Elevated xylanase activity under alkaline pH and high temperatures has been observed in various *Bacillus* species, including *Bacillus amyloliquefaciens*, *Bacillus circulans*, *Bacillus halodurans*, *Bacillus pumilus*, *Bacillus stearothermophilus*, and *B. subtilis*. Moreover, thermotolerant xylanase producers have been identified among species such as *Clostridium thermocellum*, *Kluyveromyces lactis*, *Rhodothermus marinus*, and *Thermotoga thermarum* [17,63].

Xylanase genes can also be sourced from bacterial consortia. In this study, for instance, a metagenomic approach enabled the discovery of the xylanase XynM1. Biochemical characterization showed that the optimal temperature and pH for XynM1 activity are 80 °C and 7, respectively [64].

Therefore, xylanases are regarded as a broadly distributed class of enzymes among living organisms from diverse ecological environments. As a result, extensive possibilities arise for the discovery of xylanases with a desirable set of characteristics, including high catalytic activity, stability under high- and low-temperature conditions, and resilience to specific pH levels.

## 5. Production of Xylanase

Industrial application of xylanases demands enzymes with specific attributes, including stability over broad pH and temperature ranges, high specific activity, and resistance to inhibitory metal ions and chemicals. Additional requirements encompass cost-efficiency, environmental safety, and ease of operation. Most native xylanases, however, do not naturally possess all these properties, limiting their industrial utility. Native enzymes often exhibit low yields and are incompatible with standard fermentation workflows, making large-scale production difficult [2]. Furthermore, isolating pure enzymes from natural sources presents significant challenges due to the complex mixture of proteins produced during microbial cultivation, which necessitates multiple purification steps and increases overall costs. Thus, recombinant DNA technology stands out as a strategic approach, enabling large-scale, cost-effective, and high-purity expression of target enzymes in diverse host systems, thereby meeting the stringent demands of industrial processes [3].

### 5.1. Heterologous Expression in Bacteria

*Escherichia coli* is the most widely employed host for recombinant protein expression [2]. The success of this platform is primarily attributed to factors such as the availability of a broad range of cloning vectors, rapid bacterial growth, well-characterized genetics, physiology, and metabolism, as well as the low cost of growth media and the simplicity of transformation procedures. Additionally, *E. coli* efficiently accumulates high levels of heterologous proteins within its cytoplasm [2,3,65]; however, biomass disintegration and subsequent protein purification from a complex mixture present a complicated task for large-scale production. However, a significant limitation for commercial enzyme production using *E. coli* is safety-related concerns, given its lack of GRAS status [66]. This fact, however, is generally not an issue when we speak about industry areas not related to human or animals, such as biofuel production.

Other constraints of this system include the frequent occurrence of rare codons when used for heterologous gene expression and the necessity for specific post-translational modifications, such as disulfide bond formation and glycosylation [67]. Consequently, previous studies have reported that xylanase genes often cannot be functionally expressed in *E. coli* because they require N-glycosylation, which *E. coli* cannot perform; only simple O-glycosylation is possible [3]. Nonetheless, successful overexpression of certain recombinant xylanases in *E. coli* has been documented [68,69,70], confirming that *E. coli* can be an effective host for xylanase production [3].

It is also important to consider that recombinant proteins expressed in *E. coli* tend to form inclusion bodies within the cytoplasm. To mitigate this, several strategies can be implemented, such as modulating the rate of protein synthesis, co-expressing chaperone genes to assist proper folding, or enhancing secretion into the periplasm. Controlling the protein synthesis rate can be achieved by modifying promoter strength, fusing the target gene to other functional domains, or by optimizing cultivation conditions, including pH and temperature [3]. Nevertheless, *E. coli* tend to accumulate more protein when in insoluble form and the protein in the bodies is less subjected to protease degradation. Given the easiness of initial inclusion body purification, this may turn into an advantage if one managed to develop an effective protocol to extract target protein from the bodies.

Heterologous protein expression systems utilizing *Lactobacillus* spp. and *B. subtilis* have become attractive alternatives to *E. coli*, often achieving higher production levels [67]. Both *B. subtilis* and *Lactobacillus* are Gram-positive bacteria capable of N-glycosylation [2]. Their widespread use in industry and research is primarily attributed to their non-toxic nature and classification as GRAS [67]. Unlike *E. coli*, members of the *Bacillus* genus lack endotoxins (lipopolysaccharides), which simplifies downstream purification processes by reducing endotoxin contamination [2].

### 5.2. Heterologous Expression in Yeast

Yeasts achieve high cell densities and secrete proteins into the fermentation medium, making them suitable hosts for xylanase expression. In addition, yeast systems enable eukaryotic post-translational modifications, offering advantages over bacterial platforms [17,67]. The two most commonly employed yeast expression systems are *Saccharomyces cerevisiae* and *Komagataella phaffii* (formerly *Pichia pastoris*). The ability of yeast to secrete recombinant proteins simplifies downstream processing, facilitates easier purification, and reduces production costs by avoiding toxic intracellular accumulation of heterologous proteins [66]. At present, there is an extensive toolkit of genetic engineering strategies for the heterologous production of recombinant proteins. This includes a variety of promoters, secretion factors, and chaperones that are instrumental in boosting the expression levels of the desired protein.

*S. cerevisiae* is a well-characterized eukaryotic model organism extensively utilized for heterologous protein production, conferring it with GRAS status [71,72]. Its expression system closely resembles that of higher eukaryotes, enabling post-translational modifications and secretion of target proteins. Additionally, *S. cerevisiae* exhibits tolerance to high osmolarity, low-pH environments, and various inhibitory compounds, facilitating cost-effective and straightforward fermentation processes that support rapid cell growth under both aerobic and anaerobic conditions; this is an advantage in industrial applications [66]. Despite these benefits, limitations such as inefficient secretion, protein misfolding, hyperglycosylation, and abnormal proteolytic processing can hinder its effectiveness as an expression system [66,72].

*K. phaffii* is widely utilized for heterologous protein production and, along with *S. cerevisiae*, holds GRAS status [66]. This yeast offers many advantages characteristic of higher eukaryotic expression systems, including efficient protein processing, proper folding, and posttranslational modifications, while maintaining the ease of genetic manipulation comparable to *E. coli* or *S. cerevisiae*. Its application is generally faster, more straightforward, and more cost-effective than baculovirus or mammalian systems, often resulting in higher protein yields [73].

Compared to *S. cerevisiae*, *K. phaffii* provides increased heterologous protein yields, can reach higher cell densities, and uniquely utilizes methanol as the sole carbon source. These features confer benefits in protein maturation, particularly in folding and glycosylation, which positively influence enzyme properties and thermal stability [66]. As a yeast species, *K. phaffii* shares genetic manipulations with *S. cerevisiae* but offers substantially higher expression levels—up to 100-fold—making it an increasingly valuable system for recombinant protein production [73]. Another major difference between these two yeasts is that the homologous recombination pathway in *K. phaffii* is significantly weaker than the non-homologous end joining pathway [74].

However, limitations in secretion efficiency, especially related to membrane translocation, signal peptide cleavage, and endoplasmic reticulum folding processes, pose challenges to maximal yield. Genetic engineering strategies targeting these bottlenecks have emerged as critical approaches to optimize product secretion and stability [66]. The review [66] provides detailed insights into practical and technological solutions for improving recombinant protein expression in *K. phaffii.* Notably, due to its low endogenous protein secretion, *K. phaffii* efficiently produces and secretes heterologous proteins at high yields, thereby reducing purification costs.

In summary, yeast-based expression systems look very attractive for industry as they demonstrate high growth rate, growth in simple, cheap media, compatibility with industrial bioreactors (due to robust cell structure) and thus the ability to easily scale-up processes. Another helpful feature is that yeasts are prone to secrete highly N-glycosylated proteins, which is needed often precisely for active xylanase production, as mentioned before. Finally, yeast biomass may be used as a valuable animal feed additive instead of wasting (when antibiotic resistance markerless strains are used).

In industrial production, *K. phaffii* looks more suitable than *S. cerevisiae*. *K. phaffii* has stronger promoters (e.g., pAOX1, pGAP), higher secretion ability and can grow up to higher cell densities, thus increasing productivity further; their ability to secrete a large amount of simplified protein is higher and they are able to utilize methanol as a sole carbon and energy source.

On the other hand, yeasts secrete active proteases, which decrease the stability of the target product. In the case of *K. phaffii*, methanol is often used, which is a toxic and highly flammable substance.

### 5.3. Heterologous Expression in Filamentous Fungi

Filamentous fungi are proficient producers of xylanases, facilitating both heterologous and homologous gene expression, often achieving high yields through their native promoters [75]. These fungi have been extensively engineered to enhance protein secretion capacities, making them suitable platforms for the functional expression of foreign xylanases derived from diverse sources [2]. Notably, species such as *Trichoderma reesei*, *Aspergillus kawachi*, and *A. niger* have been employed for xylanase production [17].

From a practical point of view, their remarkable feature is their ability to use wastes containing plant biomass as a substrate. Although filamentous fungi are traditionally used as industrial heterologous xylanase producers due to their ability to produce and secrete large amounts of soluble proteins, they have some shortcomings. Genetical manipulations are impaired due to the lack of well-established methods and these organisms are not characterized as being as good as yeast and bacteria when employed in this role. Their fragile mycelia are easily damaged during agitation in submerged fermentation while solid-state fermentation (traditionally used for filamentous fungi) has a lot of drawbacks itself (e.g., difficulties controlling parameters due to substrate heterogeneity, challenges in scaling up and complex downstream processing). Finally, filamentous fungi capable of producing xylanase, being natural plant biomass destructors, usually produce cellulases, which is undesirable for some areas including the paper and textile industry.

### 5.4. Heterologous Expression in Plants

In comparison with native xylanase, transgenic plants display greater enzyme stability and simplified purification methods. Expression of recombinant xylanase in *Arabidopsis* has been successfully accomplished, with the highest accumulation during flowering stages. Additionally, *C. thermocellum* xylanase has been expressed in genetically modified rice and tobacco plants [17].

Production in plants can be easily upscaled, as plants are essentially natural bioreactors. Productivity of such systems is low, however.

### 5.5. Xylanase Purification

Enzymes are essential biomolecules with a broad range of applications in industry and biomedicine. Therefore, the purification of these biomolecules is a crucial prerequisite for their effective utilization in industrial processes. Crude preparations (raw culture broth, supernatant) contain secondary metabolites and numerous other proteins, including cellulases, β-glucosidases, mannanases, proteases, and others. Only after purification can the enzyme’s molecular mass be accurately measured, its pI determined, the N-terminal sequence or amino acid composition analyzed, and its affiliation to a particular CAZy family confirmed.

The purification of xylanase from various microorganisms began in 1977 [76]. Decades of practical experience in purification procedures have led to the development of a relatively universal protocol comprising the following stages: ammonium sulfate precipitation followed by dialysis for enzyme concentration and removal of certain impurities; gel filtration on Sephadex G-25 for size-based separation and elimination of low-molecular-weight contaminants; subsequent purification employing anion- and cation-exchange resins at optimal pH values to enhance purity and selectivity [77,78,79].

This procedure has been used for purifying xylanase from various sources. For example, [80] applied DEAE 52 and fast protein liquid chromatography on CM Sepharose Fast Flow columns to purify xylanase produced by *Streptomyces rameus* L2001. Study [81] described purification of a 70-kDa endoxylanase from *Penicillium purpurogenum* through an ammonium sulfate fractionation, gel filtration on Bio-Gel P10, DEAE-cellulose, and CM-Sephadex chromatography.

In some cases, ammonium phosphate is employed for protein salting-out, as referenced in [82]. The phosphate ion occupies a different position in the Hofmeister series relative to sulfate. Although sulfate ions can precipitate proteins more completely, phosphate ions are more selective, inducing the precipitation of mainly more hydrophobic proteins while leaving more hydrophilic enzymes (such as GH) in solution. Ethanol is less frequently used for precipitation; similar to isopropanol, it allows rapid concentration, does not introduce ions that could interfere with subsequent purification steps, and may partially denature contaminating proteins while preserving the activity of the target enzyme. However, alcohols have low selectivity and pose a risk of denaturation and activity loss when the optimal concentration or temperature is exceeded (> 10 °C).

An example of alcohol precipitation was described by [83], where crude enzyme from *Myceliophthora heterothallica* was precipitated with 96% ethanol and further purified using two-step chromatography: gel filtration (Sephadex G-75) followed by anion-exchange chromatography (Resource™ Q).

However, traditional methods for purifying xylanase enzymes, as mentioned above, have several drawbacks, including high labor intensity, elevated costs, and low protein yields. To address these issues, a one-step liquid–liquid fractionation method using aqueous two-phase systems (ATPS) was developed [84]. This approach is based on partitioning enzymes between two immiscible aqueous phases formed by mixing polyethylene glycol (PEG) with an inorganic salt—most commonly (NH_4_)_2_SO_4_ or sodium phosphate—at specific concentration ratios. At a pH of around 6–7 and temperatures of 4–10 °C, xylanases typically preferentially distribute into the PEG-rich upper phase, retaining up to 90% of their original activity. Phase separation is achieved via centrifugation (3000–5000 g, 10–15 min), followed by enzyme recovery from the PEG phase through dialysis or ultrafiltration. This method offers gentle conditions that prevent enzyme denaturation, along with high activity recovery while simultaneously removing low-molecular-weight impurities and colored metabolites [85,86].

The advantages of the purification method within a ATPS compared to traditional purification techniques include low energy consumption coupled with high yield, the use of more cost-effective materials, enhanced resolution, and environmental preservation. Due to these benefits, the method has been applied in practice. In [87], the authors employed this technique for purifying alkaline xylanase from *Aspergillus candidus*. Study [88] described purification *Bacillus subtilis* xylanase using an alcohol-salt-based ATPS, while in [89,90] the authors utilized PEG/salt ATPS systems for isolating xylanase from *Trichoderma harzianum* and *Aspergillus oryzae* LC1, respectively.

It is also noteworthy that partial purification of proteins can be achieved through adsorption onto hydroxyapatite at pH 5.5–6.5, followed by desorption using a phosphate buffer [91].

Microporous activated carbon can play a significant role in the purification of xylanase and xylooligosaccharides by functioning as a selective adsorbent for the removal of colored impurities (such as lignin) and inhibitors (such as furfural, hydroxymethylfurfural, and acetic acid) from hydrolysates, thereby enhancing the purity and yield of the desired products [92].

In addition to traditional PEG/salt systems, alternative approaches based on salt-free mixtures of PEG and dextran are also investigated. In recent years, ion-liquid and biphasic systems have gained attention, offering even higher selectivity and environmental safety processes [79,93].

For example, new xylanases have recently been discovered, isolated, and purified. Two novel xylanolytic enzymes, a xylanase and a β-xylosidase, were simultaneously isolated and characterized from the extracellular medium of *Byssochlamys spectabilis* ATHUM 8891 (anamorph *Paecilomyces variotii* ATHUM 8891), grown on Brewer’s Spent Grain as a sole carbon source. They represent the first pair of characterized xylanolytic enzymes of the genus *Byssochlamys* and the first extensively characterized xylanolytic enzymes of the family *Thermoascaceae*. In contrast to other xylanolytic enzymes isolated from the same family, both enzymes are characterized by exceptional thermostability and stability at low pH values, in addition to activity optima at temperatures around 65 °C and acidic pH values. Applying nano-LC-ESI-MS/MS analysis of the purified SDS-PAGE bands, we sequenced fragments of both proteins. Based on sequence-comparison methods, both proteins appeared conserved within the genus Byssochlamys. Xylanase was classified within Glycoside Hydrolase family 11 (GH 11), while β-xylosidase in Glycoside Hydrolase family 3 (GH 3). The two enzymes showed a synergistic action against xylan by rapidly transforming almost 40% of birchwood xylan to xylose. The biochemical profile of both enzymes renders them an efficient set of biocatalysts for the hydrolysis of xylan in demanding biorefinery applications [94].

It is important to distinguish between the production of preparations from natural hosts and recombinant hosts, as concerns primarily relate to the residual presence of marker genes, which are typically antibiotic resistance genes. Such preparations are potentially hazardous to produce. Currently, this issue is addressed through the effective removal of antibiotic resistance marker genes from the genomes of producer strains [66].

## 6. Protein Engineering of Xylanases: Strategies and Applications

**Figure 5:**
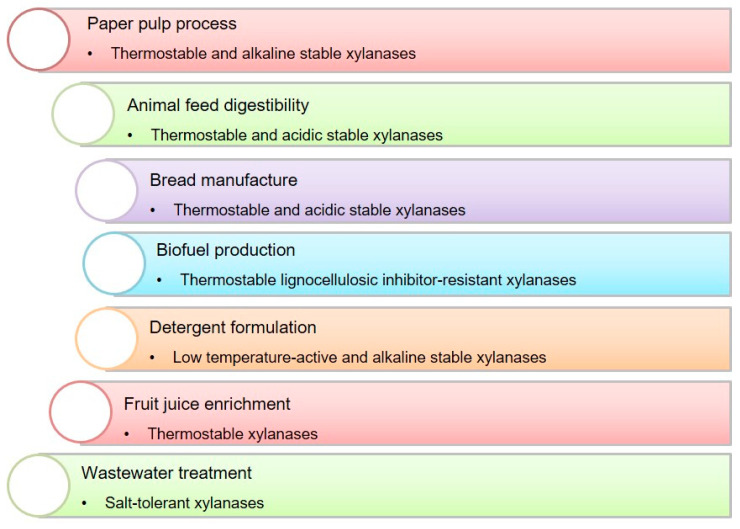
Xylanases and their desired characteristics for various industrial processes. Adapted from [95].

The modification of xylanases through protein engineering implies targeted alterations of their amino acid sequences to develop enzymes with improved or new functional characteristics. These include enhanced stability under extreme pH and temperature conditions, increased thermostability, and elevated catalytic activity. Methods such as rational design, semi-rational design, and directed evolution are commonly employed to obtain xylanase variants adapted for diverse industrial applications [95]. Xylanases and their characteristics for some industrial processes are shown in Figure 5.

### 6.1. Directed Evolution

Directed evolution imitates natural evolutionary processes within a laboratory setting, serving as a powerful strategy to enhance protein activity and stability [95,96]. The approach involves iterative cycles of generating genetic diversity followed by screening or selection. Unlike natural evolution, which primarily aims for survival and reproduction, directed evolution employs intentionally elevated mutation and recombination rates to derive variants with desired functions. The typical workflow consists of two key steps: (1) gene diversification through random mutagenesis and/or recombination to create a broad library of variants and (2) high-throughput screening or selection to isolate variants exhibiting improved phenotypes. Successful variants serve as templates for subsequent cycles, progressively refining protein properties through iterative selection [97].

The primary goal of directed evolution is to engineer proteins, particularly enzymes, to meet specific industrial needs or to create entirely novel functional capabilities [98].

Numerous directed evolution techniques have been developed, encompassing both recombinant-based approaches, such as DNA shuffling, fragment shuffling, and Random CHImeragenesis on Transient Templates PCR, and non-recombinant-based approaches, including error-prone Modification-PCR and sequence saturation mutagenesis [97]. For instance, the authors of [99] employed the xylanase XynHBN188A derived from HBP8. Directed evolution was used to increase the enzyme’s activity (reaching 3986.7 U/mg, a 2.8-fold increase compared to the WT) and to substantially enhance its pH stability.

### 6.2. Rational Design

Unlike directed evolution [100], which mimics natural evolutionary processes to optimize protein stability and activity through high-throughput screening, rational design instead relies on detailed, atom-level insights into protein structure–function relationships [101]. Progress in computational technologies—such as three-dimensional structure prediction, multiple sequence alignments, and molecular dynamics modeling—has increased the effectiveness of rational design approaches and markedly reduced the size of mutation libraries required for exploration [102]. These methods are broadly applicable, fast, and can, in principle, be translated into algorithms that quantitatively predict the efficacy of designed sequences [103]. However, incomplete knowledge of enzyme catalytic mechanisms and protein structure–function coupling may limit the precision and success rate of rational design strategies [100].

We consider two examples of applying a rational design strategy to improve xylanase properties. In [102], a GH11 family xylanase was enhanced via rational design. The introduction of two disulfide bonds improved structural stability and activity, which in turn resulted in a 75% increase in activity, a 12.1 °C rise at melting temperature (T_m_), and an 80-fold increase in half-life relative to the WT enzyme. It was further shown that introducing two additional mutations (Q125A/I129S) increased catalytic activity by 30% by improving active-site dynamics.

Another study improved the catalytic performance of *B. circulans* xylanase (Bcx) via rational, structure-based design. Systematic analysis of flexible motions demonstrated that the Bcx residue R49 limits the conformational changes required for substrate binding and participates in flexibility modulation. Site-saturation mutagenesis at R49 produced mutants offering a choice between flexibility and rigidity. The most active mutant, R49N, displayed superior catalytic characteristics, including a 7.51-fold increase in efficiency, enhanced conformational stability, and increased accumulation of xylose oligomers (2.18-fold more xylobiose and 1.72-fold more xylotriose) [104].

### 6.3. Semi-Rational Design

Semi-rational enzyme engineering combines rational design principles with directed evolution techniques to fit enzyme properties [105]. In semi-rational engineering, extensive employment of bioinformatics and computational algorithms is crucial for the identification of mutational sites. These tools aid in selecting amino acid replacements and predicting optimal target positions [95]. The approach typically involves structural analysis of the enzyme’s 3D conformation, focusing on key regions such as the catalytic pocket, substrate-binding sites, or access tunnels [96]. Mutations are often introduced at the same amino acid positions across different variants, allowing for the generation of a small but highly informative library [106,107]. This method reduces the extensive screening burden of purely random directed evolution while offering greater flexibility than strictly rational design approaches [108].

The Combinatorial Active-site Saturation Test is a frequently employed semi-rational strategy. This method involves identifying key amino acid regions from structural information and subjecting them to combinatorial saturation mutagenesis. The combination of mutations can produce synergistic, positive effects on enzyme function. Although such combinatorial approaches historically resulted in large, unwieldy libraries, the advent of advanced computational methods now facilitates the creation of small, intelligent libraries. This progress allows for the prior elimination of destabilizing mutations that are detrimental to proper protein folding [109].

Numerous studies have employed the aforementioned methods to engineer xylanases with improved thermal tolerance, pH stability, and catalytic activity [95].

### 6.4. Improving Xylanase Alkaliphility

Xylanases exhibiting robust activity under alkaline pH and elevated temperatures are highly desirable for applications in the paper pulp and detergent industries [95,110]

However, most microbial-derived and commercially available xylanases operate optimally under slightly acidic or neutral conditions. Although xylanases from *Bacillus agaradhaerens* and *Bacillus* sp. 41M-1 have demonstrated activity in alkaline environments, their activity rapidly declines under such conditions [110]. Consequently, enhancing the alkaliphilicity of xylanases is of great importance [95].

Protein engineering serves as a powerful approach to improve specific enzyme features, including increasing alkaliphility—manifested as enhanced alkaline stability and a shifted optimal pH toward higher values—and to identify key amino acids responsible for these properties. The enzyme’s surface residues play a crucial role in adaptation to alkaline conditions; previous studies have shown that surface-exposed charged amino acids influence conformational stability via noncovalent interactions such as electrostatic forces and hydrogen bonds, as well as interactions with water molecules. Alkaliphility has been improved by decreasing the number of acidic amino acids and increasing the number of arginine residues [95]. Modifying the ratio of negative to positive surface residues—specifically, reducing negative and increasing positive residues—correlates with enhanced alkaliphility [111]. The most successful strategy for adapting xylanases to alkaline environments involves the addition of arginine residues to polar surface regions, which enhances enzyme stability and activity under high-pH conditions [110].

### 6.5. Improving Xylanase Thermostability

The high thermostability and elevated temperature optimum are essential for the widespread use of xylanases. Thermostability can be improved by incorporating additional disulfide bonds, enhancing hydrogen bonds, and modifying the enzyme surface [112,113,114]. Among these, the introduction of extra disulfide bonds is a common and effective approach to enhance enzyme thermostability [115].

The main impact of this approach is to stabilize the enzyme by decreasing its flexibility. Several studies have observed that incorporating disulfide bonds into xylanase, particularly at the N-terminus or in α-helical regions, increases intramolecular interactions and effectively enhances the overall stability of the protein [116,117]. In other words, the stability of xylanase at elevated temperatures depends substantially on the proper folding of the N-terminus and α-helix segments. However, the precise interactions between the N-terminus, the α-helix, and the β-sheet core remain incompletely understood [114]. In [118], two disulfide bonds were employed to connect the N-terminus and the α-helix with the β-sheet core. These bonds stabilized the protein structure by reducing entropy in the unfolded state. Specifically, in the Xyn2 mutant, two disulfide bonds independently linked the N-terminus and α-helix to the β-sheet core. As a result, the half-life of Xyn2C14–52, as well as mutant Xyn2C59–149, was extended at 60 °C, suggesting that establishing connections between the N-terminus, α-helix, and β-sheet core contributes to overall protein stability. Furthermore, these mutants retained higher residual activity after incubation at 70 °C for 10 min compared to the WT enzyme. Notably, the mutants showed significantly reduced residual activity after treatment with 10 mM dithiothreitol at 4 °C for 12 h relative to untreated mutants under similar conditions, with the WT enzyme exhibiting negligible change.

In other studies, where additional disulfide bonds were introduced and an increase in the thermostability of xylanase was observed, it was reported that reducing the flexibility of the linker region enhanced overall rigidity [119], as well as increased surface pK_a_ values and hydrogen bonding interactions that contribute to the stabilization of the N-terminal structure [110]. Furthermore, stabilization of flexible, non-catalytic regions was also noted as a contributing factor [102].

Another strategy for enzyme stabilization involves modifying the N- or C-termini, as unstructured terminal regions can destabilize the enzyme and potentially hinder substrate access to the active site. Consequently, the removal or engineering of these residues can enhance both the stability and catalytic efficiency of xylanases [120]. Several studies have confirmed that modifications to the N- or C-terminus lead to increased thermostability in these enzymes [120,121,122].

Another approach to enhance enzyme stability involves site-specific amino acid substitutions [123,124,125,126]. Studies have reported that these modifications can lead to stabilization of the N-terminus and the active site, as well as an increase in surface positive charge and hydrophilicity [126]. Additionally, such substitutions have been shown to stabilize the most thermolabile regions of the enzyme [125].

It is also noteworthy that descriptive studies of the structures of thermophilic and mesophilic proteins have revealed a higher surface content of arginine in thermophilic proteins. These thermophilic proteins demonstrated greater stability at elevated temperatures, which may be attributed to their increased arginine content [127].

Strategies to improve the thermostability of enzymes also involve the substitution of specific amino acid residues, notably glutamic acid and proline. For example, research outlined in [101] identified amino acids associated with thermophilic properties. Thermostable xylanases with maximal activity temperatures of 80 °C or higher commonly contain proline and glutamic acid residues at certain positions. For example, a mutant enzyme, V81P/G82E/D185P/S186E, was designed by replacing the corresponding residues in the XynAS9 enzyme. Specifically, the mutant exhibited a shift in its optimal temperature by roughly 17 °C upward and an over ninefold increase in the half-life during thermal inactivation at 70 °C.

### 6.6. Improving Xylanase Catalytic Performance

The catalytic performance—including catalytic efficiency and activity—of enzymes is a fundamental property critical to numerous biotechnological applications. Numerous studies have focused on engineering GH11 xylanases to enhance their catalytic performance. However, the underlying factors governing the catalytic properties of GH11 xylanases remain insufficiently understood. It is recognized that amino acid substitutions that augment enzymatic function can occur either within the active site or at distal sites outside the catalytic center [95].

As demonstrated in [128], site-directed mutagenesis within the active site resulted in a 20% increase in enzymatic activity. The authors hypothesize that this enhancement arises from improved amino acid interactions, the establishment of supplementary hydrogen bonds, and an expanded spatial configuration, thereby promoting substrate accessibility and efficient product dissociation.

Modifications outside the active site can enhance catalytic efficiency by inducing structural changes in the catalytic region, often through the formation of new non-covalent interactions between the substituted residues and catalytic amino acids, or with other amino acids interacting with the catalytic residue [95]. Additionally, conformational dynamics—such as altered flexibility in the thumb and loop regions [102,129], as well as changes in hydrogen bonding, van der Waals forces, and hydrophobic contacts—play a crucial role in regulating enzymatic activity [130].

Modifications and truncations at the N- or C- terminus can also influence catalytic activity [122,131]. In [122], molecular docking analyses revealed that the hydrogen bonding networks between the subsites of the truncated variant of the xylanase gene (28C) and xylohexaose differed from those of the WT gene (Mtxylan2), indicating alterations in substrate–subsite interaction patterns. In another investigation [131], molecular dynamics simulations demonstrated that mutations W6F/Q7H and N8Y led to an increase in the volume and surface area of the active site cleft, thereby facilitating substrate entry and product release, which ultimately enhanced the enzyme’s catalytic efficiency.

Additionally, it has been reported that sometimes the formation of disulfide bonds not only enhances thermal stability but also increases catalytic activity [115,119]. In [119], the authors suggest that this effect is associated with conformational changes at the active site and prolonged interactions between catalytic amino acids and the substrate.

Table 1 presents various studies in which the mentioned xylanase properties were improved using protein engineering techniques.

## 7. Conclusions

This analysis highlights the considerable potential of xylanases in diverse industrial fields, from pulp and paper and food industries to biofuel production. The effective utilization of these enzymes is grounded in a comprehensive understanding of their fundamental characteristics, as outlined in this review.

It has been demonstrated that the structural diversity of xylan directly influences the specificity and catalytic mechanisms of various xylanase families, such as GH10 and GH11. Inhibition analysis methods offer valuable insights into the architecture of active sites and the catalytic processes of these enzymes.

Although native producers undoubtedly represent an important source of enzymes, the extraction and purification of xylanases from these native sources often present significant challenges, including low yields and complex downstream processing. Therefore, the selection of an optimal producer is critical for obtaining commercially valuable preparations. To overcome the limitations of WT strains, recombinant expression systems are increasingly employed as a strategic solution, enabling high-yield production and superior purity of the target enzyme.

Protein engineering constitutes a highly promising approach for the rational enhancement of xylanase properties, including increased thermostability, resistance to alkaline pH, and elevated specific activity. Consequently, this facilitates the production of heterologous enzymes tailored for a broad range of conditions. Resent progress in artificial intelligence development makes computational tools much more effective. Machine learning algorithms trained on sequence and structure databases are able to predict structure-stabilizing mutations and changes in protein properties. Combining AI-assisted structure prediction with high-throughput screening is likely to be a way to modify enzymes faster and more precisely.

## Figures and Tables

**Table 1:** Summary of effective protein engineering approaches for xylanase improvement.

Xylanase Gene	Microorganism Source	Producer Strain	Genetic Engineering	Effect Observed	Reference	Suggested Cause
CFXyl3 (GenBank accession No. WP_013115499.1)	*Cellulomonas* *flavigena*	*E. coli* BL21 (DE3)	Modification of the N-terminus	The optimal temperature was increased by 30 °C, and the melting point was increased by 34.5 °C	[121]	Replacement of the N-terminus with that of a thermostable xylanase
GH11 xylanase Mtxylan	*Myceliophthora thermophila*	*E. coli* BL21 (DE3)	C-terminal truncation	The catalytic activity was increased by 9.3 times; the optimal temperature was increased by 5 °C	[122]	Decreased flexibility of the C-terminus, an enlarged binding pocket, and alterations in the hydrogen bonding network between the cleft subsites and the substrate
GH11 xylanase XynLC9	*B. subtilis*	*E. coli* BL21 (DE3)	Amino acid substitutions at the N-terminus	The catalytic activity was increased by 2.6-fold, and thermostability was also improved	[131]	Mutations resulted in an increased volume and surface area of the active site cleft, thereby facilitating substrate entry and product release and subsequently enhancing the enzyme’s catalytic efficiency
GH11 xylanase XynASP	*Aspergillus* *saccharolyticus* JOP 1030-1	*E. coli* BL21 (DE3)	Introduction of additional disulfide bonds.	The half-life at 50 °C increased 130.9-fold, and the catalytic efficiency increased 9.3-fold.	[119]	Reducing the flexibility of the cord region boosted the overall rigidity, resulting in improved thermal stability. The extensive catalytic cleft and prolonged contact between catalytic residues and the substrate were likely key factors in enhancing catalytic activity. Maintaining the thumb highly flexible can offset the negative impact on catalytic activity during the thermal stability modification of the cord region
Endo-1,4-β- xylanase Bcx	*B. circulans*	*E. coli* BL21 (DE3)	Amino acid substitution	The catalytic efficiency increased 7.51-fold	[104]	The R49 component of Bcx (1) constrains the global conformational changes essential for substrate binding and (2) is involved in modulating flexible motion
Mesophilic Xylanase PjxA (GenBank accession No MK948222)	*Penicillium* *janthinellum* MA21601	*E. coli* BL21 (DE3)	Modification of the N-terminus	The melting temperature (T_m_) value was improved from 21.3 ℃ to 76.6 °C, and its half-life at 60 ℃ increased 107-fold	[132]	The thermostability of PjxA was improved through the introduction of a disulfide bond (T2C-T29C) between the irregular loop and β-strand A2. Additionally, mutations T2C, T29C, N30L, and Y15F contributed to increased hydrophobicity at the N-terminus
Xylanase XT6	*Geobacillus* *stearothermophilus*	*E. coli* DH5α	Directed evolution	Turnover number (*k*_cat_) and catalytic efficiency (*k*_cat_/*k*_M_) increased 13- and 6.5-folds respectively	[133]	Mutations are located within βα-loops. Conformational alterations of these loops may facilitate loop movement, thereby promoting product release and ultimately enhancing catalytic efficiency
GH11 Xylanase, *Ev*Xyn11	Not stated	*E. coli* XL1-Blue MR	Directed evolution	The T_m_ approximately increased by 25 °C	[123]	T13F may increase hydrophobic interactions, and S9P may apparently lock the conformation of a surface loop
Xylanase XynR8	Neocallimastigales rumen	*E. coli* BL21 (DE3)	Random point mutagenesis combined with site-directed mutagenesis	At 65 °C, the WT enzyme completely inactivated after 30 min of incubation, whereas XynR8_VNE retained approximately 65% of its activity under the same conditions	[124]	The obtained amino acid substitutions are located within the N-terminus, core, and α-helix domains, respectively. Therefore, the stability of these three regions may be critical for the thermostability of family 11 xylanases
*Orpinomyces* XynA xylanase	*Orpinomyces* sp. PC-2	*E. coli* BL21-CodonPlus (DE3)-RIPL	Modification of the N-terminus	The specific activity was increased	[120]	The presence of unstructured amino acids at the N-terminus contributes to the destabilization of xylanases and potentially reduces substrate accessibility to the active site. Consequently, removal of these residues may enhance enzyme stability and catalytic activity
Xylanase AnXynB (NCBI accession No. ACA24724)	*A. niger* ATCC1015	*E. coli* BL21 (DE3)	Amino acid substitutions	The catalytic activity increased by 72% and the thermostability was improved	[134]	The increasing binding affinity of enzyme and substrate
Xylanase XynCDBFV (GenBank accession No. KP691331)	*Neocallimastix patriciarum*	*K. phaffii strain X33*	Amino acid substitutions	The catalytic activity increased by 20%. The optimal temperature decreased by 5 °C	[128]	The activity-enhancing mutations affect active-site residues. Authors propose that phenylalanine at these positions maintains stacking interactions while creating extra space for substrate entry and product release. Phe-163 likely does not contact the substrate directly but stacks with conserved Tyr-111, which hydrogen-bonds to the +1 sugar
Acidic Xylanase PjxA	*P. janthinellum* MA21601	*E. coli BL21 (DE3)*	Amino acid substitutions	The half-life at 50 °C increased 115.11-fold, the specific activity increased 2.02-fold	[126]	Stabilization of the N-terminus and the active center of PjxA, the increase in surface positive charge and hydrophilicity are the main reasons for the improved thermostability and catalytic activity of PjxA
GH10 Xylanase (Xyn30Y5)	*Bacillus* sp. *30Y5*	*E. coli* BL21 (DE3)	Amino acid substitutions	The specific activity increased 2.9-fold, the catalytic efficiency increased 2-fold, stability after incubation at 60 °C for 30 min was enhanced, and an optimal pH shifted from 7.0 to 8.0	[125]	The flexibility of α5 helix and loop7 may be crucial to affect the catalytic activity. And the increase in stability of the most unstable regions including loop3, loop6, loop7, α7 helix and N/C-terminal regions was vital for thermostability improvement
GH-11 Xylanase	*Thermobacillus xylanilyticus*	*E. coli* JM109-DE3	The introduction of disulfide bonds	The half-life at 70 °C was increased 10-fold and the specific activity is almost doubled	[115]	The introduction of disulphide bonds by directed mutagenesis is a straightforward strategy to improve thermostability
Xyn12.2 Xylanase	The termite gut symbiont metagenome	*E. coli* Rosetta (DE3)	The introduc-tion of disulfide bonds	The bagasse hydrolysis at pH 9.0 and 60 °C increased 2–3-fold	[110]	Formation of an exterior disulfide bond, increased surface *pK_a_*, and hydrogen bonds for stabilizing the N-terminal random structure are key determinants for improved catalytic activity under conditions of increased temperature and pH
GH30 Xylanase	*Dickeya dadantii* DCE-01	*E. coli BL21 (DE3)*	Directed evolution	The optimal pH value decreased and acid–base tolerance improved. The enzymatic activity increased 1.6-fold	[129]	Mutation is positioned on the protein surface within the catalytic domain, distal from the active site. The other two mutations are situated within loop regions. The increased flexibility of these loop regions in the mutant may contribute to the observed enhancement in enzymatic activity
A hybrid xylanase gene, atx (GenBank accession No. AY949844)	Synthetic construct	*K. phaffii* GS115 (his4)	Directed evolution	The xylanase activity in the culture supernatant was increased 2.92-fold	[130]	Proline possibly produced weaker hydrogen bond, van der Waals force and hydrophobic interaction with other residues nearby than leucine, especially for V174, contributing to the flexibility of catalytic residue E177
GH11 xylanase XynII (GenBank accession No. CAA49293.1)	*T. reesei*	*E. coli* BL21 (DE3)	The introduc-tion of disulfide bonds	The enzymatic activity increased by 75%, the Tm increased by a 12.1 °C	[102]	Incorporation of additional disulfide bonds stabilized flexible non-catalytic regions, and the Q125A/I129S mutations in the thumb region enhanced catalytic dynamics
GH11 Xylanase (UniProt: accession No. P55330)	*A. niger*	*E. coli* BL21 (DE3)	Amino acid substitutions	The enzymatic activity increased 1.5-fold and the thermostability was enhanced	[135]	The coordinated effect of the mutations
GH11 xylanase XynA	*S. rameus L2001*	*E. coli* BL21 (DE3)	Amino acid substitutions	The optimal temperature shifted from 60 °C to 80 °C. The residual enzyme activity remained above 85% when incubated at 80 °C and 90 °C for 12 h. The specific activity and catalytic efficiency were improved	[136]	Reducing the flexibility of amino acid residues in the loop region, promoting a more compact xylanase structure, and increasing the surface net charge are partly responsible for the increased thermal stability of the mutants

## Data Availability

The authors confirm that the data supporting the findings of this study are available within the article.

## Abbreviations

The following abbreviations are used in this manuscript:

ATPS	Aqueous two-phase systems
CAZy	Carbohydrate-Active Enzymes
Cryo-EM	Cryo-electron microscopy
GH	Glycosyl hydrolases
GRAS	Generally Recognized as Safe
LCB	Lignocellulosic biomass
NMR	Nuclear magnetic resonance
T_m_	Melting temperature
PEG	Polyethylene glycol
SAXS	Small-angle X-ray scattering
pI	Isoelectric point
TAXI	*Triticum aestivum* xylanase inhibitor
XOS	Xylooligosaccharides
XIPs	Xylanase Inhibitor Proteins
WT	Wild-type
